# Intranasal Drug Administration in Alzheimer-Type Dementia: Towards Clinical Applications

**DOI:** 10.3390/pharmaceutics15051399

**Published:** 2023-05-03

**Authors:** Raquel Taléns-Visconti, Jesus Vicente de Julián-Ortiz, Ofelia Vila-Busó, Octavio Diez-Sales, Amparo Nácher

**Affiliations:** 1 Department of Pharmacy and Pharmaceutical Technology and Parasitology, Faculty of Pharmacy, University of Valencia, Av. Vicent Andrés Estellés s/n, 46100 Valencia, Spain; octavio.diez@uv.es (O.D.-S.); amparo.nacher@uv.es (A.N.); 2 Molecular Topology and Drug Design Research Unit, Department of Physical Chemistry, Faculty of Pharmacy, University of Valencia, Av. Vicent Andrés Estellés s/n, 46100 Valencia, Spain; 3 Colloids Research Unit, Department of Physical Chemistry, Faculty of Pharmacy, University of Valencia, Av. Vicent Andrés Estellés s/n, 46100 Valencia, Spain; ofelia.vila@uv.es; 4 Instituto Interuniversitario de Investigación de Reconocimiento Molecular y Desarrollo Tecnológico (IDM), Universitat Politècnica de València, Av. Vicent Andrés Estellés s/n, Burjassot, 46100 Valencia, Spain

**Keywords:** intranasal delivery, Alzheimer, blood–brain barrier, nanoparticles, intranasal devices, nanostructured lipid carriers

## Abstract

Alzheimer-type dementia (ATD) treatments face limitations in crossing the blood–brain barrier and systemic adverse effects. Intranasal administration offers a direct route to the brain via the nasal cavity’s olfactory and trigeminal pathways. However, nasal physiology can hinder drug absorption and limit bioavailability. Therefore, the physicochemical characteristics of formulations must be optimized by means of technological strategies. Among the strategies that have been explored, lipid-based nanosystems, particularly nanostructured lipid carriers, are promising in preclinical investigations with minimal toxicity and therapeutic efficacy due to their ability to overcome challenges associated with other nanocarriers. We review the studies of nanostructured lipid carriers for intranasal administration in the treatment of ATD. Currently, no drugs for intranasal administration in ATD have marketing approval, with only three candidates, insulin, rivastigmine and APH-1105, being clinically investigated. Further studies with different candidates will eventually confirm the potential of the intranasal route of administration in the treatment of ATD.

## 1. Introduction

The general objective of this work is to carry out a review on the intranasal administration of drugs in the treatment of Alzheimer’s disease and related dementias. This review aims to offer a critical analysis of the most promising trends in nose–brain administered lipid nanoparticles that are likely to yield therapeutically useful outcomes, highlighting the most relevant and significant findings based on current knowledge in the field. To this end, technological strategies will be identified to overcome the limitations associated with this route of administration, as well as the most promising type of formulation for drug access to the brain from the nasal cavity. Finally, the current situation of the intranasal route of administration in the treatment of Alzheimer’s disease and related dementias is evaluated.

### 1.1. The Pathophysiology of Alzheimer-Type Dementias

Alzheimer-type dementia (ATD) is a condition of the central nervous system (CNS) characterized by cognitive and behavioral deterioration that is usually associated with age, with a long and progressive course that ultimately leads to the death of the patient [1]. It is estimated that 60–80 percent of dementia cases are caused by ATD [2,3]. There were over 50 million ATD cases globally as of 2020 [4].

Dementia is more common in some parts of the world than others, with a prevalence of 4.7% in Central Europe and an 8.7% prevalence in North Africa and the Middle East [5,6]. For example, in Spain, the prevalence of dementia in the population over 65 years of age is between 4% and 9%, and is higher in the female sex [2], and the seventh most common cause of mortality in the USA is currently ATD [7]. Although up to 10% of cases have an early beginning and affect people in their 30s to mid-60s, adults over 65 years old account for the majority of cases [7,8]. Around 6% of adults 65 and older are affected, with women being more frequently affected than males [9]. Between 1990 and 2019, the incidence of Alzheimer’s disease and other dementias increased by 147.95% worldwide [4]. This disease imposes a significant financial burden on society, with a global cost estimated to be 1 trillion euros per year [4].

**Figure 1:**
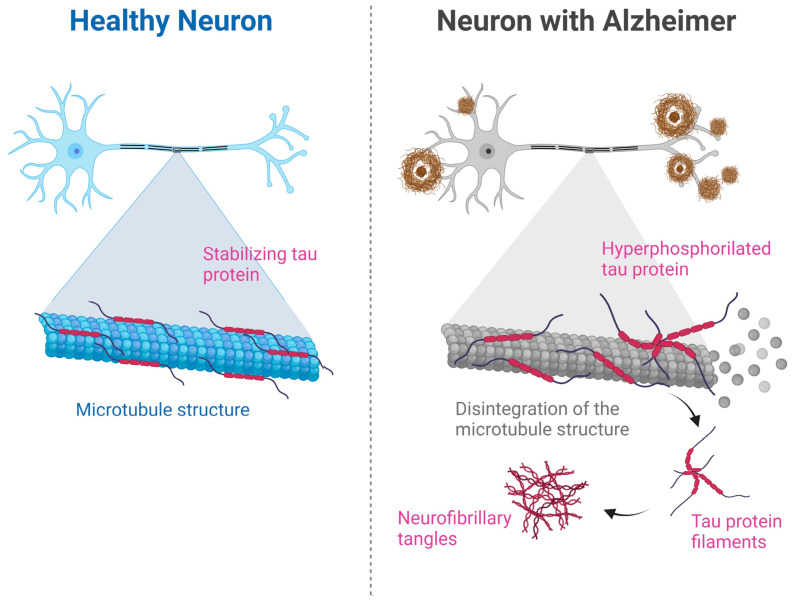
Main differences between a healthy brain and a brain with Alzheimer-type dementias.

The disease is named Alzheimer’s disease in honor of Alois Alzheimer, a German psychiatrist and pathologist, who in 1906, first described the case of a 50-year-old woman with presenile dementia [10]. This disease is characterized by the progressive and irreversible damage and destruction of nerve cells, which leads to brain atrophy and death of neurons. The exact pathophysiological cause is not yet confirmed, although it is believed to be a multifactorial disorder. It occurs due to a series of physiological changes, including the formation of ß-amyloid plaques, tau hyperphosphorylation (a protein that is part of the cytoskeleton of neurons), mitochondrial dysfunction, neuronal excitotoxicity, cholinergic imbalance, neuroinflammation, oxidative stress and other factors. Various hypotheses have been put forward to explain the pathogenesis of ATD, including the cholinergic hypothesis, the tau hypothesis and the amyloid cascade hypothesis. In addition, among the main biomarkers of ATD are the deposition of the ß-amyloid peptide, which leads to the formation of ß-amyloid plaques, and the hyperphosphorylation of tau, which leads to the formation of neurofibrillary tangles [11]. Figure 1 shows the main differences between a healthy brain and the brain of a patient with ATD.

### 1.2. Current Treatment of Alzheimer-Type Dementias

This complex aetiology poses a great challenge to finding a potential therapy that allows treating or correcting abnormal physiological conditions. Currently, the drugs approved by the FDA to treat the cognitive symptoms of ATD are based on the modulation of neurotransmitters or enzymes, among which are the following: acetylcholinesterase (AChE) inhibitors: donepezil [12,13], galantamine [14] and rivastigmine [15,16]; and the receptor antagonist N-methyl-d-aspartate also known as memantine [17]. However, none of these drugs can prevent the progression of the disease, but instead act by temporarily increasing cognitive functions through a partial improvement of cholinergic and glutamic neurotransmission [18].

In June 2021, the FDA approved the monoclonal antibody Aducanumab for the treatment of ATD [19]. However, the EMA denied marketing authorization for this drug because the results of the main studies were conflicting, and the drug was not shown to be sufficiently safe and effective [20].

As seen in Table 1, the available treatments for ATD use generally the peripheral route for the administration of drugs. The main drawback of peripheral administration is the limited accessibility of the drug to the brain from the blood due to the presence of a safety barrier that restricts the entry of molecules into the brain: the blood–brain barrier (BBB). At the same time, hepatic first-pass metabolism and enzymatic degradation (in the case of oral administration) and systemic clearance (in the oral and parenteral routes) also significantly reduce the bioavailability of the drug. In addition, systemic administration may present other limitations such as binding to plasma proteins, low volume of distribution, delay in drug delivery to the brain through the blood, and systemic adverse effects, especially at the gastrointestinal level (such as nausea and vomiting) [21].

Therefore, there is great interest in identifying new alternative routes of administration within order to overcome the limitations of those currently available, increase the bioavailability of drugs at the site of action and obtain a better benefit/risk ratio in treatments.

In this context, nasal administration offers an alternative and novel approach for direct drug access to the brain.

### 1.3. Barriers That Prevent Drug Access to the Brain

There are two physiological barriers that the drug must overcome to reach the brain: the blood–brain barrier (BBB), which is an important limiting factor, and the blood–cerebrospinal fluid barrier, which separates the cerebrospinal fluid (CSF) systemic circulation and protects it from exposure to any exogenous substance that may be toxic.

The BBB is a complex structure made up of endothelial cells sealed by tight junctions, pericytes, basement membrane and astrocytes that create a continuous barrier [24]. The BBB separates brain tissue from the peripheral circulation allowing selective access of necessary nutrients and hormones to the CNS and, in turn, restricts the entry of foreign substances or neurotoxins, including drugs. In addition, it is responsible for maintaining an optimal ion composition for neuronal signaling [25].

Small lipophilic molecules and certain essential nutrients can pass through the blood–brain barrier (BBB) via passive diffusion (both paracellular and transcellular pathways) or through active transport mechanisms [26].

**Figure 2:**
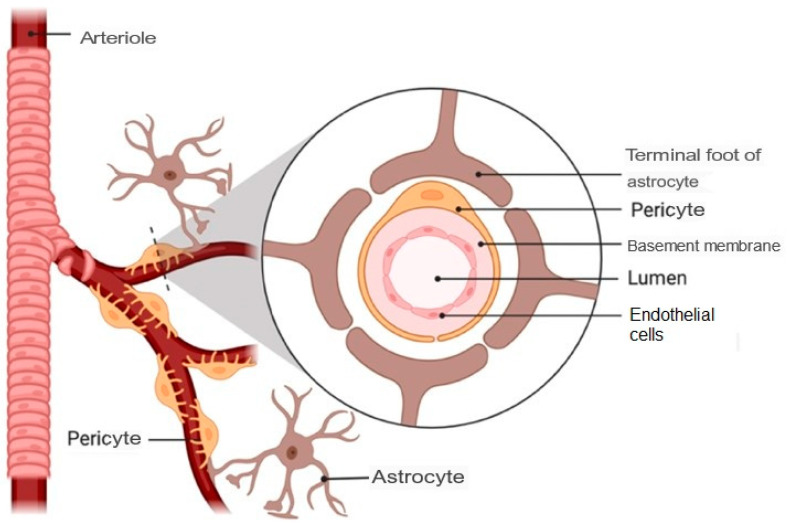
Structure of the blood–brain barrier.

Molecules that can pass through the BBB via passive diffusion usually need to meet specific criteria, such as being lipid-soluble, having no electrical charge at physiological pH, having a molecular weight lower than 500 Da and presenting a partition coefficient between 0.5 and 6.0 [27]. In Figure 2, the structure of the BBB is shown.

### 1.4. Intranasal Route for Drug Administration

#### 1.4.1. Nasal Cavity Anatomy

The main functions of the nasal cavity are breathing and smell, and, in addition, it exerts a protective action of filtering, warming and humidifying the inhaled air before it reaches the lower respiratory tract [28]. Likewise, it is composed of two symmetrical cavities that are divided by the nasal septum and lined with a layer of mucosa. These cavities in turn are divided into three regions:

- Vestibular region: This is the most anterior area, and contains nasal hairs to filter inhaled particles and the main cell type is squamous epithelial cells. In this region, drug absorption is very limited [29].
- Respiratory region: This is the area with the largest surface (about 130 cm^2^) and the most vascularity, which makes it an ideal place for the systemic absorption of drugs. It encompasses the lateral walls of the nostrils, including the protruding nasal turbinates. There are four main cell types: goblet, ciliated, non-ciliated columnar and basal. In addition, the respiratory region is innervated by the maxillary and ophthalmic branches of the trigeminal nerve (V1, V2) [30].
- Olfactory region: This is located on the roof of the nasal cavity and covers only 10% of the total nasal area. There are different types of cells: basal cells, olfactory nerve cells, support cells, cilia and trigeminal neurons. In addition, the olfactory region facilitates the transport of drugs to the brain having a direct connection with the brain through the olfactory and trigeminal neurons [31].

**Figure 3:**
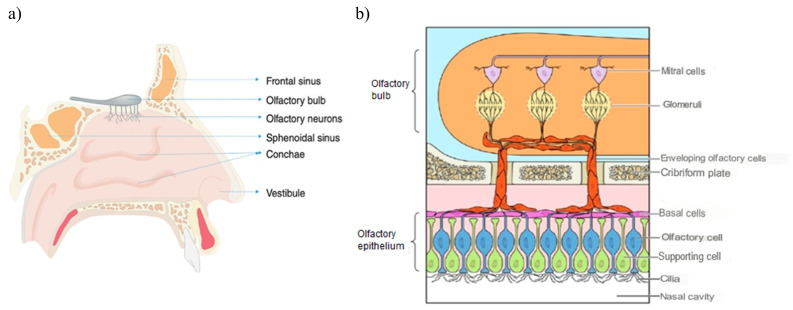
(**a**) Anatomy of the nasal cavity, adapted from [32]. (**b**) Schematic representation of the olfactory region, adapted from [33].

Figure 3 illustrates the anatomy of the nasal cavity and provides a schematic representation of the olfactory region.

#### 1.4.2. Intranasal Absorption

The first step in drug absorption from the nasal cavity is to cross the mucus layer. Mucin, which is the major mucus protein capable of binding solutes, hinders drug diffusion. Non-ionized, lipophilic and small particles easily pass through this layer of mucus. Thus, the molecular weight and lipophilicity of drugs can have a large impact on the rate and degree of nasal absorption of drugs. However, other physicochemical properties of the drug and the formulation must also be considered, as well as some physiological factors of the nose, which may also influence the bioavailability and transport of the drug from the nose to the brain [34].

**Figure 4:**
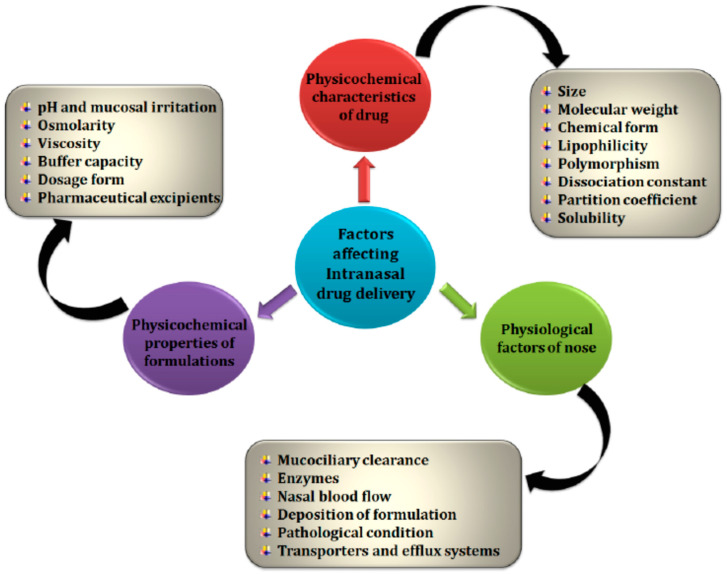
Factors affecting absorption through the nasal route [34].

Figure 4 summarizes the main factors that influence the nasal absorption of drugs administered by this route.

**Figure 5:**
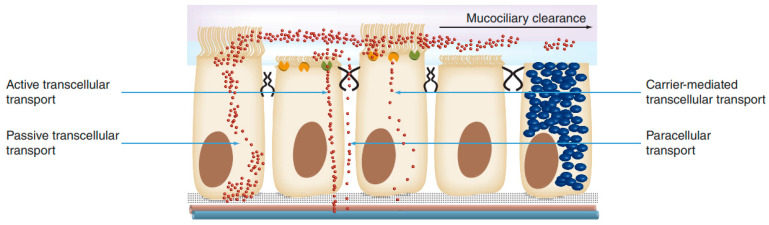
Mechanisms of drug absorption across the nasal epithelium [32].

Once through the mucus, different mechanisms of absorption can be followed through the nasal mucosa: transcellular diffusion, paracellular transport, endocytic vesicle-mediated transport and carrier-mediated transport. Transcellular diffusion is the main mechanism of absorption and is the most suitable for lipophilic and low molecular weight molecules [26,35]. Figure 5 schematically represents the different mechanisms of absorption through the nasal mucosa.

#### 1.4.3. Nose–Brain Absorption Pathways

(a) Direct Absorption Mechanism

- Olfactory Sensory Neurons

The olfactory nerve pathway is a direct and primary route of drug transport from the nasal cavity to the brain, preventing its passage through the BBB. When the drug reaches the olfactory receptors after passing through the nasal mucosa, there are two transport mechanisms to the brain: the intracellular route, in which the drug is taken up by the olfactory neurons through a process of endocytosis, and the extracellular route, in which the drug crosses the nasal epithelium through the tight junctions between the olfactory nerves and the supporting cells [36].

**Figure 6:**
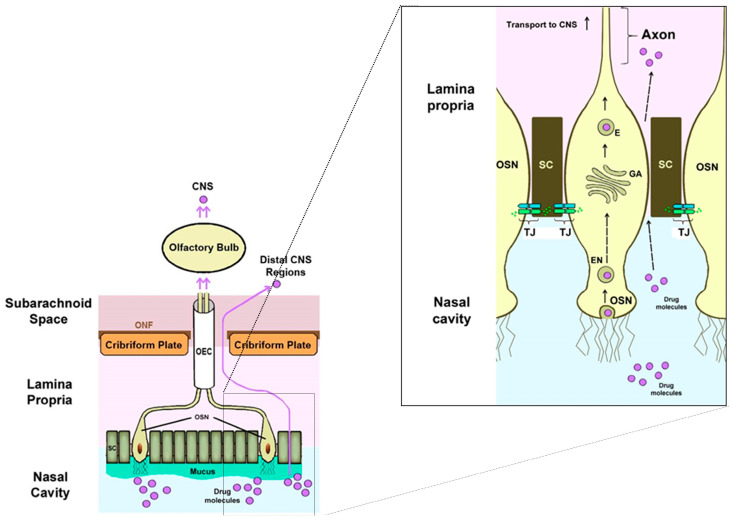
Intranasal transport of drugs through the olfactory route to the CNS by intracellular and extracellular routes [36].

In both cases, the molecules travel through the cribriform plate, which separates the brain from the nasal cavity, to enter the olfactory bulb and the CSF. Subsequently, the drug can be distributed from the CSF to the brain by mixing with the interstitial fluid of the brain. The intracellular route requires from hours to days for the active fraction to reach the different regions of the brain and is adequate for the passage of lipophilic molecules, while the extracellular route only requires a few minutes for the active fraction to reach the brain and is suitable for the passage of hydrophilic molecules [37]. Figure 6 schematically represents the intranasal transport of drugs through the olfactory route to the CNS by the intracellular and extracellular route.

- Trigeminal Neurons

**Figure 7:**
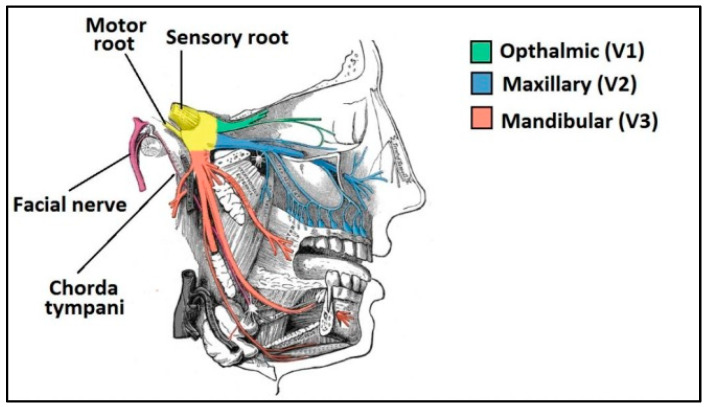
Innervation of the trigeminal nerve in the nasal cavity [39].

Both the respiratory and olfactory epithelium are supplied with trigeminal nerves (Figure 7). Branches from the ophthalmic division (V1) of the trigeminal nerve innervate the dorsal and anterior part of the nasal mucosa, and those from the maxillary division (V2) innervate the turbinates.

Once the drug has diffused through the mucosa, it allows its transport directly to the brainstem at the level of the pons, avoiding its passage through the BBB. Transport through the trigeminal route can also occur intracellularly or extracellularly, as occurs with olfactory neurons [38].

(b) Indirect Absorption Mechanism: The Systemic Route

When a drug is deposited in the nasal cavity and depending on its residence time, a part of it can be absorbed and enter the systemic circulation due to the rich vasculature of the respiratory epithelium and reach the brain through the BBB. This is a secondary route of drug transport to the brain after nasal administration [40].

Small lipophilic molecules gain access to the bloodstream and penetrate the BBB much more easily than high molecular weight and hydrophilic molecules. In addition, the active substances that use this route undergo renal and hepatic metabolism, so that the amount of drug that finally reaches the brain is very low and depends on the partition coefficient (0.5–6.0) and the molecular weight of the drug (<500 Da). It also leads to a higher incidence of unwanted side effects in other peripheral organs [41].

**Figure 8:**
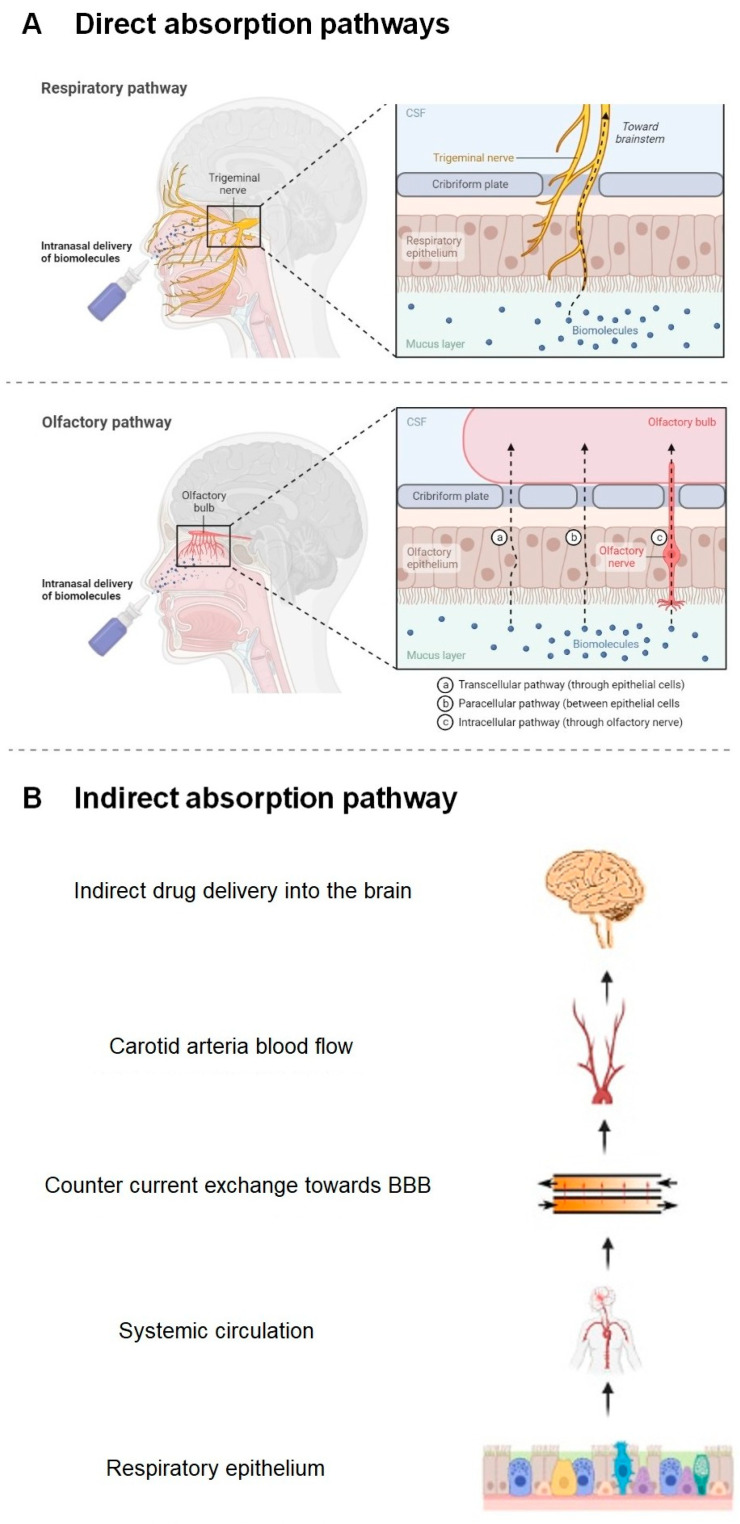
Schematic representation of the structure of the nasal cavity and the mechanisms of drug absorption through the nasal mucosa to the brain. (**A**) Direct absorption mechanism through olfactory nerve and trigeminal route. (**B**) Indirect absorption mechanism by systemic route [42].

Figure 8 schematically represents the structure of the nasal cavity and the different mechanisms of drug transport through the nasal mucosa to the brain.

#### 1.4.4. Advantages and Disadvantages of the Nasal Route

To date, numerous techniques have been developed to circumvent the BBB and improve drug delivery to the brain, including invasive techniques, such as surgical exposure or intrathecal and intraventricular routes of administration.

The intranasal route offers a non-invasive strategy to bypass the BBB and provide direct drug access to the brain in a short period of time, as it has a direct connection to the brain through neural channels. In addition, the nasal cavity has a large number of blood vessels with rapid blood flow and has a large available surface area for absorption. For this reason, it offers other advantages such as rapid drug absorption, avoidance of hepatic metabolism and easy administration, which increases patient compliance [43].

However, there are several obstacles that can limit absorption of drug through this pathway, such as poor drug permeability from the nasal mucosa, mucociliary clearance, enzymatic degradation of the drug, low drug residence time and possible toxicity that the formulation or drug can produce on the nasal mucosa. In addition, due to the limited volume of application through this route, the dose of the drug may be limited depending on its solubility in the vehicle, so it will only be applicable to potent drugs that allow obtaining sufficient pharmacological effect at low doses or by using dry powders. Likewise, it would be convenient to reserve this route for those active principles with a wide therapeutic margin due to the possible inaccuracy of the dosage after administration. All these characteristics make it difficult to obtain bioavailability into the brain via the intranasal route [29]. For this reason, numerous technological strategies have been investigated to overcome these drawbacks and improve the bioavailability of drugs administered intranasally, which will be detailed later.

Table 2 indicates the main advantages and disadvantages associated with the administration of drugs through the nose.

## 2. Materials and Methods

To carry out this work, a bibliographical review of scientific articles on the access of drugs to the brain through intranasal administration in the treatment of ATD was carried out, as well as strategies to improve absorption and available nanotherapeutic strategies. In addition, the website of the Spanish Agency for Medicines and Health Products (AEMPS) and the website of the European Medicines Agency (EMA) and the Food and Drug Administration (FDA) were consulted for those drugs that have been approved for the treatment of Alzheimer-type dementias. Likewise, the Clinical trials.gov website was consulted to search for existing clinical trials of intranasal administration in ATD.

Search keywords used were: “Alzheimer’s disease” AND (“intranasal drug delivery” OR “nose-to-brain delivery” OR “intranasal devices”) AND (“blood–brain barrier” OR “brain targeting” OR “nanoparticles” OR “lipid nanoparticles” OR “NLC” OR “intranasal devices” OR “nano-lipidic formulation”).

Finally, the Biorender^®^ website was used to create some of the figures used in the work (https://biorender.com, accessed from July 2022 to April 2023).

## 3. Results and Discussion

In this section, possible strategies for overcoming the limitation of nasal administration are shown. Moreover, the importance of new drug nanocarriers are discussed, indicating some of the more relevant actives that are likely to be clinically used for the intranasal treatment of Alzheimer-type-dementia. To do this, the aforementioned search keywords were used.

### 3.1. Possible Strategies for Overcoming the Limitations of Nasal Administration

The following are possible strategies that can be applied to overcome the obstacles that the nasal route presents to the delivery of drugs to the brain.

#### 3.1.1. Systems That Reduce Mucociliary Clearance

Mucociliary clearance acts as a defense mechanism of the respiratory tract that allows the effective and rapid elimination of inhaled hazardous substances through the combined effect produced by the mucus and the cilia of the columnar cells, which trap and drag the particles to the gastrointestinal system to be swallowed. When a formulation is administered via the nose, it has to go through mucociliary clearance, which will eliminate a part of the drug and which, together with the short residence time of the drug in the nasal cavity, will limit its absorption through the mucosa [44].

To avoid rapid clearance and improve drug residence time in the nasal cavity, bioadhesive systems formulated with mucoadhesive polymers, viscosifiers, nasal gels that are generated in situ in response to certain stimuli (e.g., temperature, ionic strength, pH), hydrogels, etc., which favor nasal absorption of drugs as a result of increased residence time in the area of administration, can be used [45]. Another strategy is to provide the particles with a positive surface charge, for example, with stearylamine, to increase mucoadhesiveness in the nasal cavity by interacting with the negatively charged sialic acid of the mucous layer. Table 3 lists several examples of mucoadhesion-enhancing excipients that have been explored for the development of intranasal drug delivery systems. Among them, the most widely used have been chitosan and cyclodextrins, due to their low toxicity and absorption-promoting effect.

In addition, the use of adequate administration devices that allow deep access of the drug to the olfactory region, associated with head tilt, can minimize mucociliary clearance, favoring greater circulation of the drug to the brain. Several delivery devices have been developed (listed in Table 3), of which only the Optinose^®^, Precision Olfactory Delivery^®^ and ViaNase^®^ devices have been used in studies of intranasal administration to the brain in humans [46]. The ViaNase^®^ device was used in the clinical trial of intranasal insulin delivery for the treatment of ATD [47]. Likewise, although some of these devices, such as the Optinose^®^, have been approved by the FDA [48] for the treatment of migraine, none of them have been authorized for nasal administration in ATD.

#### 3.1.2. Systems That Reduce Enzymatic Degradation

The epithelial barrier and the lumen of the nasal cavity contain some metabolic enzymes such as cytochrome P450 (CYP450) isoforms, transferases, carboxyesterases and peptidases, responsible for the degradation of various drugs administered through the nasal cavity, especially proteins and peptides [49].

These enzymes affect the bioavailability and integrity of the drug when metabolizing it, which could be overcome through the use of enzyme inhibitors, prodrugs and new drug carriers, which will be specified later. In Table 3, numerous examples of each of these strategies are presented.

Among the prodrugs that have been studied, Gln-1062 (MEMOGAIN^®^), a lipophilic prodrug of galantamine, stands out. A randomized double-blind study was conducted in subjects aged ≥65 years in which some cognitive improvement was observed, minimizing complications associated with oral administration of galantamine, such as syncope and bradycardia. However, some adverse effects related to the gastrointestinal tract and nasal mucosal irritation, both dose-dependent, were reported, which were the cause of withdrawal in some subjects [50]. Moreover, it has not been approved by the FDA, nor are there any current studies related to this prodrug.

#### 3.1.3. Systems That Reduce Irritation of the Nasal Mucosa

The irritation of the nasal mucosa is a critical parameter that must be taken into account during the development of a formulation. When deposited in the nasal cavity, the formulation comes into contact with the intact mucosa. Most drugs and excipients can exert some toxic effect, irritation or damage to the mucosa, which can range from mild sneezing to skin rash, itching and even severe or systemic toxicity. Toxicity is often exacerbated by the use of polymers, mucoadhesive agents or any other novel approach that can dramatically increase the contact time of the mucosal layer with the drug to increase its absorption across the nasal epithelium [51].

To minimize the incidence of nasal toxicity, the use of drugs, polymers or surfactants physiologically compatible and non-irritating to the nasal mucosa is recommended. In addition, the pH, isotonicity and viscosity of the formulation must be taken into account during drug development to prevent irritation of the mucosa.

Apart from this, the mode of administration of the drug, the size of the droplets or the particle size if using dry powder also has a significant impact on toxicity. Administration by nasal drops achieves maximum exposure to the respiratory region, directing the drug to the systemic circulation and to the pulmonary organs, while the administration of the formulation with the head inclined upwards with suitable devices ensures maximum focus on the olfactory region, facilitating the absorption of the drug directly by the brain [52]. However, maintaining the position of the head inclined upwards is complicated and uncomfortable for the patient, which makes it necessary to develop administration devices that facilitate drug deposition in the olfactory region, such as those mentioned in Section 3.1.1.

#### 3.1.4. Systems That Promote Nasal Permeability

The nasal cavity is surrounded by a thin layer of mucus of a lipophilic nature. The presence of tight junctions in the olfactory and respiratory epithelium, together with the protective mucosal lining, act as a selective filter that delays the penetration and diffusion of drugs through the nasal mucosa. In addition, transcellular diffusion is the main transport mechanism from the nose to the brain, which mainly allows the passage of lipophilic and low molecular weight substances [40]. For this reason, the nasal route is more suitable for smaller lipophilic molecules, whereas large, polar and hydrophilic drugs, such as proteins and peptides, experience poor nasal permeability. The nasal absorption of polar drugs can be improved though the use of suitable absorption promoters and also by new drug carriers, which will be specified later. In Table 3, several examples of these strategies are shown.

### 3.2. New Drug Carriers as a Potential Strategy

Among all the strategies to improve nasal absorption that have been discussed, new nanotechnology-based drug carriers have shown the ability to overcome the resistance generated by mucociliary clearance, secretion systems and epithelial tight junctions. In addition, they allow drugs to be protected from chemical or metabolic degradation and improve solubility or transport through biological membranes, leading to better orientation and fewer systemic adverse effects [53]. For this reason, in recent years they have gained the greatest interest in research. Likewise, it is necessary to consider the appropriate selection of excipients, the physicochemical properties (particle size, size distribution, zeta potential and drug charge), biodegradability, biocompatibility, the release profile and also the safety profile [54].

Various nanotherapeutic strategies have been explored for intranasal treatment of ATD, including polymeric and inorganic nanoparticles, and lipid-based nanocarriers. Lipid-based nanocarriers are promising candidates for drug delivery from the nose to the brain in the treatment of ATD. They offer greater brain-targeting potential, rapid brain uptake, biodegradability and bioaccessibility, and overcome challenges associated with polymeric nanoparticles, such as cytotoxicity. Lipid-based nanocarriers can incorporate both hydrophilic and lipophilic drugs, provide protection and offer controlled drug release by immobilizing the drug in the solid lipid matrix, which biodegrades easily [55].

**Figure 9:**
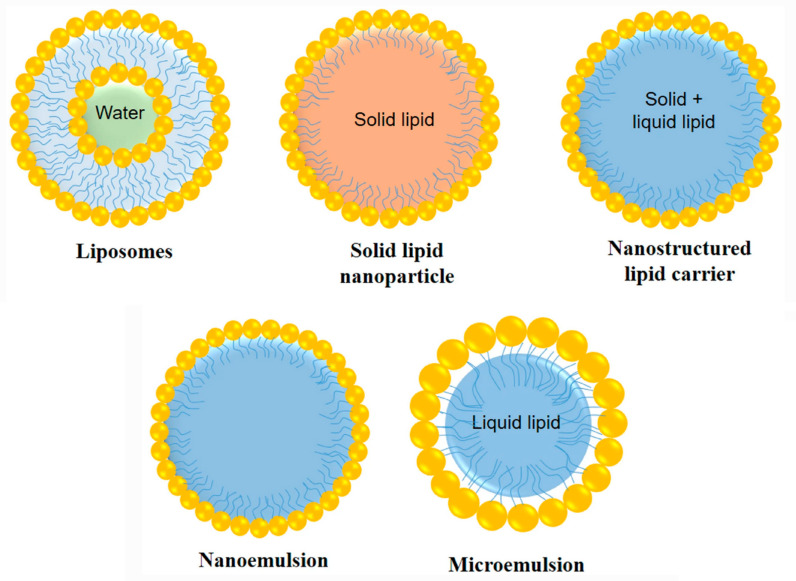
Lipid-based nanocarriers that have been explored for drug access to the brain from the nasal route [62].

There are several types of lipid-based nanocarriers, such as liposomes [56], niosomes [57,58], microemulsions [59], nanoemulsions [60] and solid-matrix lipid nanoparticles [61] as shown in Figure 9. The latter have generated greater interest in recent years due to the advantages they present and will be detailed in the following section.

#### 3.2.1. Nanostructured Lipid Carriers for Intranasal Delivery

Solid matrix lipid nanoparticles are aqueous dispersions of solid particles that may be composed of physiological lipids and stabilized by one or several emulsifiers, with an average particle size that can range between 100 and 300 nm. There are two types, solid lipid nanoparticles (SLNs) and nanostructured lipid carriers (NLCs), as seen in Figure 9.

SLNs present a lipid matrix composed of a single solid lipid that has a highly organized structure and are considered the first generation of lipid nanoparticles, while NLCs present a disorganized internal lipid matrix, formed by a mixture of solid and liquid lipids and are known as second-generation lipid nanoparticles [63].

**Figure 10:**
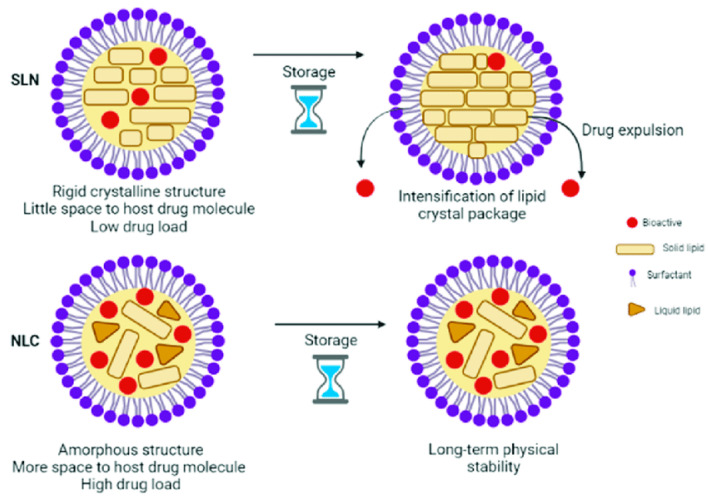
Main differences between solid lipid nanoparticles (SLN) and nanostructured lipid carriers (NLCs) [66].

In the case of NLCs, the disruption of the lipid matrix caused by the liquid lipid allows for higher encapsulation efficiency (EE), since most hydrophobic molecules show better solubility in liquid than in solid lipids, and a low expulsion of the encapsulated drug during storage, i.e., a higher stability. For this reason, current research focuses on NLCs [64,65]. Figure 10 shows the main differences between SLNs and NLCs.

Thus, NLCs have important advantages over the first generation of lipid nanoparticles. Likewise, NLCs offer advantages over other nanosystems for nasal administration, since they can be manufactured from biocompatible and biodegradable components, such as physiological lipids and other GRAS (Generally Recognized As Safe) excipients [67].

In addition, they protect drugs against enzymatic degradation, increasing the residence time in the nasal cavity and improving bioavailability. Similarly, it is possible to produce NLCs with the desired features for nasal administration to the brain, namely a mean particle size ≤ 200 nm, a polydispersity index (PDI) of 0.3 and a zeta potential (ZP) of 30 mV [68].

However, the low viscosity of NLCs may decrease the bioavailability of the drug in the brain as the residence time reduced. To overcome this drawback, the inclusion of NLC formulations in mucoadhesive materials has been described as an appropriate strategy [69].

Moreover, during storage, NLCs can aggregate, reducing their potential as controlled-release drug carriers, which can be avoided by modifying the stability of formulations through storage temperature and pH. When it comes to safety, it is important to consider that the type and amount of surfactant used in formulations can sometimes lead to sensitivity, irritation or cytotoxicity. For this reason, it is essential to develop detailed nanotoxicological studies to identify the specific elements that guarantee its safety, such as the biological compatibility of the formulation components, the effect of particle size and surface charge [42].

Different experiments have been used to evaluate pharmacokinetic parameters after the intranasal administration of NLCs in Alzheimer-type dementia compared with free drug solution or other administration routes [70]. In this sense, preclinical studies have been carried out with this type of formulation using different bioactive compounds, which have obtained promising results. Some of these studies are reflected in Table 4 and are detailed below.

##### Rivastigmine

Rivastigmine (RV) is an inhibitor of the AChE enzyme that has been shown to be useful in the treatment of ATD, which is marketed as oral capsules, oral solution and transdermal patches, as shown in Table 1. However, due to its hydrophilic nature, it exhibits poor permeability through the BBB and has a short half-life (90 min) and extensive first-pass metabolism. It has, therefore, a low bioavailability and requires frequent administration, causing the side effects of accumulation at a systemic level [71]. For this reason, researchers have tried to explore alternative administration routes that would make it possible to overcome these limitations of the formulations marketed with this drug.

In 2017, Wavikar et al. developed an in situ gelling system of rivastigmine NLCs for nasal administration using the ethanol injection method [72]. The resulting formulation showed a double improvement in drug permeability due to the mucoadhesive behavior of the gel in the upper nasal cavity. Subacute and nasal toxicity studies showed that the formulation was safe for nasal administration to the brain. Additionally, administration of the gel with RV-loaded NLCs improved pharmacokinetic characteristics and biodistribution in the mouse brain, improving cognition and memory compared to intravenous formulations and simple intranasal solutions.

In 2019, Anand et al. assessed the efficacy of rivastigmine hydrogen tartrate (RHT)-loaded lipid-based nanocarriers (NLCs) via the nasal route for treating Alzheimer’s disease-associated dementia [73]. The formulation showed favorable physicochemical properties and ex vivo diffusion studies revealed sustained release of RHT from the NLCs. In vivo studies in male and female Wistar rats demonstrated improved neuronal function, accompanied by significant enhancements in memory, learning and cognitive response, indicating the therapeutic potential for ATD treatment.

##### Pioglitazone

Various studies have shown that impaired insulin signaling in the brain is one of the main factors behind the development of ATD. For this reason, some of the antidiabetic molecules have been studied for the treatment of ATD, including pioglitazone (PIO) [74].

In preclinical studies, pioglitazone was found to be effective in significantly reducing oxidative stress, as well as improving cognition and glucose use in the brain. However, subsequent clinical trials conducted with regular doses of oral PIO showed limited success due to an inadequate drug concentration reaching the brain, as it barely crosses the BBB. To achieve a therapeutic concentration at the site of action, a higher dose of the drug would be required; however, higher doses in human volunteers are restricted due to the serious dose-dependent peripheral side effects of PIO, such as bladder cancer and peripheral edema. Therefore, to improve the bioavailability of the drug in the brain, Jojo et al. developed a formulation with NLCs loaded with PIO for nasal administration [75].

The formulation obtained had good physicochemical and mucoadhesive characteristics, which significantly increased the permeability of the drug compared to its administration in an intranasal solution. Likewise, little or no toxicity was observed in the nasal epithelium.

On the other hand, the biodistribution in the brain as well as the brain/plasma ratio obtained after intranasal administration of NLC dispersion was higher compared to intravenous or intranasal PIO solution in male Wistar rats. This would contribute to achieving therapeutic concentration at the site of action, without the need for a higher dose of drug or increased systemic adverse effects, thus becoming a potential therapeutic alternative for the treatment of ATD.

##### Resveratrol

Resveratrol is a natural compound that has antiproliferative, anti-inflammatory and antioxidant properties and exerts neuroprotective effects due to the elimination of the ß-amyloid peptide and the breakdown of the amyloid precursor protein, so it could be used for prevention and treatment of ATD, alone or combined with other active ingredients. However, it exhibits poor aqueous solubility and chemical stability, along with high first-pass metabolism, which contributes to poor bioavailability after oral administration [76].

For this reason, Rajput et al. formulated an in situ gel to incorporate resveratrol-loaded NLCs for administration from the nose to the brain [77]. The formulation had good characteristics and nasal ciliotoxicity studies demonstrated the safety of the formulation.

Pharmacokinetic studies in male Sprague Dawley rats revealed faster absorption and greater bioavailability of the drug in the brain when administered intranasally in the form of a gel loaded with NLCs compared to an oral suspension of the same active ingredient. All these would demonstrate the potential brain selectivity of the intranasal formulation.

##### Berberine

Recent research has shown that the isoquinoline alkaloid berberine (BER) may be effective in the treatment of ATD as it inhibits the formation of ß-amyloid plaques, decreases tau hyperphosphorylation and reduces oxidative stress [78]. To precisely target BER to the brain in order to maximize therapeutic effects and minimize potential adverse effects, Abo El-Enin et al. developed a formulation of BER-loaded chitosan-coated NLCs for intranasal administration [79]. Chitosan (CTS) is a mucoadhesive polymer that also acts as an absorption promoter, thereby enhancing drug penetration through the nasal mucosa and reducing mucociliary clearance.

The BER-CTS-NLCs obtained presented good physicochemical characteristics (PS < 200 nm and ZP > +30; see Table 4), and, in addition, showed prolonged release behavior and enhanced drug permeability through the nasal mucosa of sheep. Furthermore, histopathological evaluation indicated that the BER-CTS-NLCs system was safe for nasal administration.

Pharmacokinetic and brain/plasma ratio studies in male Wistar rats showed that those treated intranasally with BER-CTS-NLCs had significantly higher drug levels in the brain compared to intranasal BER solution. In Table 4, the most important aspects of the aforementioned preclinical studies are summarized.

##### Astaxanthin

Astaxanthin (AST) is a second-generation antioxidant with anti-inflammatory and neuroprotective properties and could be a promising candidate for Alzheimer’s disease (AD) therapy, but is shows poor oral bioavailability due to its high lipophilicity [80].

Shehata et al. (2023) [81] prepare and evaluate AST-loaded nanostructured lipid carriers (NLCs) for enhanced nose-to-brain drug delivery to improve the therapeutic efficacy in a rat model of AD. The Korsmeyer–Peppas model was the best fitted for AST release from NLCs and the release mechanism involves diffusion and matrix erosion.

Intranasal treatment of AD-like male albino rats with the optimized AST-NLCs improved the nasal permeability and nose-to-brain drug delivery. In fact, the rats treated with AST-NLC showed significant higher percentage of time spent in brain than AST solution. The neuroprotective role of AST against amyloid beta can be due to its potent antioxidant effects and reduction of ROS mitochondrial. In summary, AST-NLC provided significantly decreased oxidative stress, amyloidogenic pathway, neuroinflammation and apoptosis, and significantly improved the cholinergic neurotransmission compared to AST solution.

##### Donepezil

Donepezil is approved for treatment of dementia of the Alzheimer-type and is currently available in tablet form and transdermal patch in the United States [19,22,23].

Oral administration of this drug presents many drawbacks, resulting in treatment non-adherence among patients. Thus, the development of transdermal formulations for donepezil delivery is important.

Corplex™ donepezil transdermal delivery system (TDS), approved as Adlarity^®^ for the treatment of mild, moderate and severe dementia of the Alzheimer-type, is designed to deliver donepezil through the skin over a 7-day wear period and is available in 5 and 10 mg/d doses.

Butani et al. (2018) [82] developed donepezil-loaded NLCs and incorporated the optimized NLCs into the ionic-triggered gellan gum matrix for IN delivery. Pharmacokinetic study of donepezil in brain and plasma were studied (dose 1 mg/kg) in male Sprague–Dawley rats. The AUC_0–8h,brain_ of the NLC gel (IN) was 1.26-fold higher than that of the tablet (oral). This slight increase indicated that the NLC gel was not highly potential for brain targeting. However, this ratio is 1.5-fold lower in plasma, thus reducing toxicity to other organs. No PK data were provided for IV or IN administrations of the free drug. In a rat model of scopolamine-induced amnesia, the NLC gel (IN) improved cognitive function as compared with the marketed tablet (oral).

**Table 4:** Preclinical studies with nanostructured lipid carriers for intranasal administration in the treatment of Alzheimer-type dementias.

Preclinical Studies with Nanostructured Lipid Carriers					
Active Ingredient	Preparation Method	Physicochemical Properties	In Vitro Results	In Vivo Results	Reference Year
Rivastigmine	Ethanol injection method	**PS**: 123.2 nm **ZP**: 32 mV **EE**: 68.3%	Non-hemolytic, safe for intranasal administration	AUC: 506,731.3 ng.min/mL Cmax: 1984.23 ng/mL Tmax: 30 min t_1/2_: 347.65 min Increase up to 4.6–5.3 brain concentration Increased bioavailability Improves memory and cognition No or very little nasal toxicity	[72] 2017
Rivastigmine hydrogen tartrate	Emulsification/ solvent evaporation	**PS**: 266 nm **PDI**: 0.23 **ZP**: −16.58 mV **EE**: 61.8%	Controlled and sustained release of 80% of the drug for 12 h Controlled diffusion, initially through surface erosion	Significant reduction in AChE1 and AchE2 activity, which enhances neuronal function Significant improvement of memory, learning and cognitive response	[73] 2019
Pioglitazone	Microemulsification	**PS**: 211.4 nm **PDI**: 0.25 **ZP**: 14.9 mV **EE**: 70.18%	Sustained release for 24 h Fickian Diffusion Higuchi kinetics Increased permeability (1789 μg/cm^2^) No cytotoxic effect	Cmax: 1.62 μg/mL Brain/plasma ratio: 1.6 Increased bioavailability Nose–brain direct delivery	[75] 2019
Resveratrol	Ultrasonic emulsification	**PS**: 132 nm **PDI**: 0.165 **ZP**: −23 mV **EE**: 74%	Drug loading of 10 ± 3% and a mucoadhesive force of the gel of 4087 ± 115 dynes/cm^2^ Long term stability at 2–8 °C	Cmax 2.5-fold higher in brain than NLC-based in situ gel Higher concentration in the brain with the gel in situ No nasal toxicity Faster absorption	[77] 2019
Berberine	Hot homogenization and ultrasonics	**PS**: 180.9 nm **ZP**: 36.8 mV	Sustained release properties and increased ex vivo permeability through nasal mucosa	The Cbrain/Cplasma ratio at 30 min was much higher in the intranasal NLC dispersion formulation (4.56) than in the IN plain solution (2.14) or IV solution (0.26)	[79] 2022
Astaxanthin	Hot high-pressure homogenization	PS: 142.8 nm PDI: 0.247 ZP: –32.2 mV EE: 94.1%	Initial burst release followed by sustained release for 24 h AST-NLCs were stable at 4–8 °C for six months	Significantly improved the cholinergic neurotransmission compared to AST solution	[81] 2023
Donepezil	Melt emulsification- probe sonication method	PS: 112 nm PDI: 0.114 ZP: −35 mV EE: 79.25%	Drug loading of 7.9 ± 2.1% and a mucoadhesive force of the gel of 3189 ± 84.26 dynes/cm^2^	Higher drug distribution in brain (20% increase NLC vs marketed tablets) and lower drug concentration in plasma (25% reduction NLC vs tablet) NLC administration increased residence time and penetration through olfactory lobe Improved cognitive function (IN vs. oral tablet)	[82] 2018

PS: particle size; ZP: zeta potential; EE: encapsulation efficiency; PDI: polydispersity index.

These preclinical studies show that formulations with nanostructured lipid carriers have great potential to improve the bioavailability in the brain of drugs used to treat ATD through intranasal administration. Thus, all the NLCs studied present a similar line of action, since:

- They promote greater cerebral uptake than simple drug solutions, both intravenously and intranasally, leading to increased drug concentrations in the brain.
- They are more efficiently absorbed after intranasal administration and their small diameter facilitates their passage through the neuronal pathway.
- They provide a controlled and sustained release profile of the drug, unlike conventional drug delivery systems.
- They can be coated with polymers, such as chitosan, or dispersed in hydrogels, giving the formulation excellent mucoadhesive properties, prolonging the residence time in the nasal cavity and improving permeability through the nasal epithelium.
- They offer better drug residence and formulation stability during storage and greater physiological compatibility than other formulations.

### 3.3. Current Situation of Intranasal Administration in the Treatment of ATD

Preclinical studies with NLCs have shown promising results for treating ATD with low toxicity. However, there are currently no ongoing clinical trials with intranasal NLCs. One reason for this could be limitations in manufacturing methods, which require controllable, reproducible and large-scale preparation technologies. Another factor is the differences between animal and human models, which may not ensure reproducibility in humans. Finally, the lack of widely accepted international standards for regulating nanomedicines also poses a challenge [83].

Therefore, the intranasal administration of nanostructured lipid carriers for the treatment of ATD can be considered, today, as a promising alternative to currently marketed drugs, although it still requires a lot of scientific effort to put it into clinical practice.

**Figure 11:**
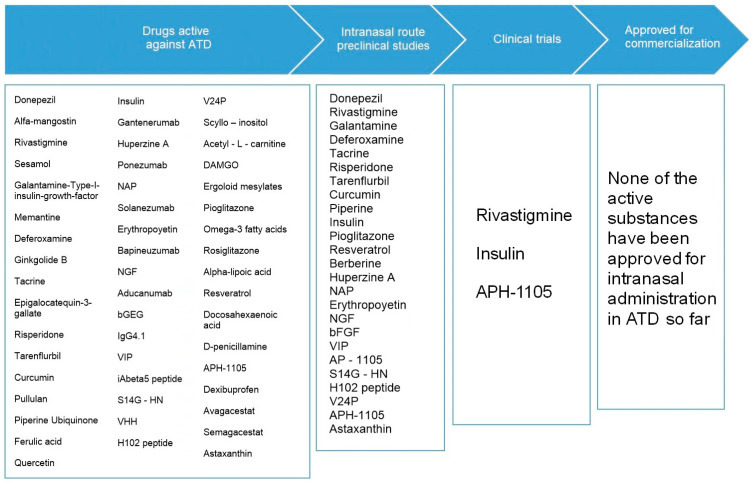
Current status of the investigation of nasal therapies for Alzheimer-type dementia.

Figure 11 summarizes the current situation in the investigation of therapies for ATD by the nasal route. As previously discussed, to date no drug has obtained marketing approval for intranasal administration in the treatment of ATD.

As can be seen, most of the trials with bioactive compounds that can be administered through the nose to treat ATD stop at their preclinical phase, and only three candidates have managed to reach the clinical investigation phase, namely insulin [84], rivastigmine [85] and APH-1105 [86].

Although no formulation for intranasal administration in ATD has yet been marketed, there is great interest in the intranasal route as an alternative administration route for the treatment of Alzheimer-type dementias, due to the advantages it entails and the very promising results that have been obtained in preclinical investigations. In the year 2016, a bioavailability and safety study was conducted by administering a unit dose of rivastigmine intravenous solution (1 mg) and nasal spray (3126 mg) to eight healthy individuals aged 58–75 years. In this study, rivastigmine nasal spray provided an effective dose for the treatment of ATD-associated dementia [87], as shown in Table 5.

Likewise, several recent studies have directly associated insulin resistance with ATD pathology, since it has been correlated with poorer cognition and higher concentrations of hyperphosphorylated tau protein. This is due to the fact that insulin influences the production and elimination of the Aβ peptide and also prevents tau hyperphosphorylation, among other effects relevant to ATD [88]. For this reason, in recent decades, clinical trials have been carried out with intranasal insulin for the treatment of ATD (Table 5).

Only one clinical trial in phase 2 with intranasal insulin is currently being carried out “SNIFF–Combo INI+EMPA” trial that began in October 2021, with an estimated completion date of October 2026 [89]. The study consists of a randomized, double-blind trial with 60 participants, in which the effects of treatment will be compared for 4 weeks with intranasal insulin (40 IU, four times daily), empagliflozin (10 mg daily) and the combination of intranasal insulin (INI) and empagliflozin (EMPA), compared with placebo, on cerebrospinal fluid (CSF) biomarkers and cognition. On the other hand, there is another clinical trial in phase 2, “SNIFF–3-Week Aptar CPS Device” trial, whose start date is expected in May 2025 and its estimated completion date is May 2029 [90]. This study will examine whether the Aptar Pharma CPS intranasal delivery device can be used in adults with preclinical ATD (i.e., cognitively normal, but with abnormal brain Aβ-peptide levels) to reliably deliver insulin or placebo four times daily for a four-week period. In addition, the effects of treatment on cognition, CSF biomarkers and cerebral perfusion will be examined. If successful, the information obtained from the study will be useful in designing future phase 3 trials of intranasal insulin, but with abnormal brain levels of Aβ peptide, to reliably administer insulin or placebo four times a day for a period of four weeks.

In addition, a clinical trial is expected to start in 2023 to assess the safety, tolerability and efficacy of intranasal administration of APH-1105 for the treatment of mild to moderate ATD [74], as shown in Table 5. The APH-1105 is a modulator of the α-secretase, an enzyme that cleaves the amyloid precursor protein into a more soluble product that is more easily cleared from the brain and does not lead to the formation of insoluble amyloid plaques.

In Table 5, both the results and the identifiers of the clinical trials that have been carried out for intranasal administration in Alzheimer-type dementias are collected.

**Table 5:** Clinical trials evaluating the intranasal administration of drugs for the treatment of Alzheimer-type dementia.

Clinical Trials for Nasal Administration in Alzheimer-Type Dementias				
Active Ingredient	Date	Phase	Results	Reference
Insulin	2007–2012	Phase 2	An improvement in memory retention and attention was observed after administration of 20 IU daily of intranasal insulin compared to placebo.	NCT00438568 [91]
Insulin	2012	Phase 2	No results have been published.	NCT01547169 [92]
Insulin	2012–2018	Phase 2	Treatment with 20 IU of insulin improved delayed memory, and both insulin doses (20 and 40 IU) preserved general cognition as assessed via the ADAS-cog score and functional abilities as assessed via the ADCS-ADL scale. There were no treatment-related serious adverse events.	NCT01595646 [47,93]
Insulin	2013–2020	Phase 2	A total of 289 participants were randomized. Among the first 49 participants, the ViaNase device was used to deliver intranasal insulin, while the rest of the participants used a second device (device 2). After one year of treatment, participants using device 2 did not show cognitive or functional benefits, while patients using the ViaNase device did show a slowdown in cognitive decline according to the ADAS-cog-12 scale and in everyday activities. No clinically important adverse events were associated.	NCT01767909 [94]
Insulin	2015–2020	Phase 2	No results have been published.	NCT02462161 [95]
Insulin	2015–2020	Phase 2	Intranasal insulin glulisine had no significant effect on improving cognition or mood; however, the number of subjects successfully enrolled and the duration of the study were limited. Intranasal insulin glulisine was relatively safe and well tolerated.	NCT02503501 [96]
Insulin	2021	Phase 2	In recruiting status.	NCT05081219 [89]
Insulin	2022	Phase 2	Not recruiting yet.	NCT05006599 [90]
Rivastigmine	2016	Phase 1	Rivastigmine nasal spray had superior absolute bioavailability compared to historical values for the oral capsule and transdermal patch determined by other investigators. It had a rapid onset of action, and a favorable safety and tolerability profile. No clinically important adverse events were associated.	ACTRN12614001313628 [87]
APH-1105	2023–2024	Phase 2	Not recruiting yet.	NCT03806478 [86]

It should be noted that among those active principles with which clinical trials have been carried out, the one that has generated the most interest would be intranasal insulin, which is the one with which the greatest number of clinical trials have been carried out and which has produced quite encouraging results.

## 4. Conclusions

The intranasal route has great potential as an alternative route of administration in the treatment of ATD, since it has numerous advantages over conventional routes of administration, among them, direct transport from the nasal cavity to the brain avoiding crossing the BBB. To obtain good bioavailability in the brain, the physicochemical characteristics of the formulations must be optimized through technological strategies (mucoadhesive polymers, prodrugs, absorption promoters, etc.) so that they are suitable for this route of transport and avoid the physiological mechanisms of elimination from the nasal cavity. Among the various technological strategies investigated to optimize intranasal formulations, lipid-based nanosystems, particularly nanostructured lipid carriers, have shown the most promise as effective systems for achieving drug access to the brain from the nasal cavity. They have generated considerable interest due to their stability, physiological compatibility and ability to overcome challenges associated with other nanocarriers. At the moment, no drugs have obtained marketing approval for intranasal administration in the treatment of ATD, and only three candidates have made it to the clinical investigation phase, namely insulin, rivastigmine and APH-1105. Further studies with different candidates will eventually confirm the potential of the intranasal route of administration in the treatment of ATD.

## Figures and Tables

**Table 1:** FDA-approved drugs for the treatment of Alzheimer-type dementias.

Active Ingredient	Route of Administration	Pharmaceutical Form	FDA Reference
Rivastigmine	Oral	Capsules Oral solution	[15,16]
	Transdermal	Transdermal patches	
Galantamine	Oral	Tablets Extended release capsules	[14]
Memantine	Oral	Tablets Oral solution Extended release capsules	[17]
Donepezil	Oral	Tablets Orodispersible tablets	[12,13,22]
	Transdermal	Transdermal patches	
Combination of memantine and donepezil	Oral	Controlled release capsules	[23]
Aducanumab	Parenteral	IV bolus	[19]

**Table 2:** Advantages and drawbacks of nasal drug administration.

Advantages	Disadvantages
Non-invasive, safe and painless Lower risk of infections Easy administration and better patient compliance Alternative to parenteral route of administration, especially for protein and peptide drugs Large absorption area and extensive network of blood and lymphatic vessels Rapid absorption and excellent bioavailability for low molecular weight drugs Prevents first-pass hepatic metabolism and drug degradation in the gastrointestinal tract Possible direct delivery to the brain bypassing the BBB via the olfactory and trigeminal nerves	Only applicable to potent drugs with a wide therapeutic margin Small volumes of administration (25–200 μL) Mucociliary clearance and low residence time Enzymatic degradation Low permeability of hydrophilic drugs Need for absorption promoters that can cause toxicity in the nasal mucosa Epithelial mucous pH 5–6.5 Interindividual variability Runny nose influences absorption Possible inaccuracy in dosage due to improper administration technique Some drugs and excipients can cause irritation to the nasal mucosa

**Table 3:** Strategies to improve nasal absorption.

Strategies to Improve Nasal Absorption					
Promoters of Absorption	Promoters of Absorption	Promoters of Absorption	Promoters of Absorption	Promoters of Absorption	Promoters of Absorption
Sodium lauryl sulfate Poloxamer Tween^®^ Span^®^ Sodium glucodeoxycholate Sodium taurodeoxycholate Taurodihydrofusidate Oleic acids Lauric acid Caprylic acid Phosphatidylcholine EDTA Citric acid Sodium salicytate Peppermint oil Cyclodextrins Chitosan Carbopol Starch Aminated gelatin	Comostat amylase Bestatin Aprotinin Amastatin Boroleucine Bacitracin Puromycin EDTA	Carboxymethylcellu-lose Microcrystalline cellulose Hydroxypropylcellu-lose Carbopol 971P Carbopol 934P Carbopol 981P Eudragrit Chitosan Cyclodextrins Stearylamine	MEMOGAIN^®^ (Gln-106	Optinase nasal device ViaNase-electronic atomizer Precision-Olfactory device Naltos device	**Polymeric nanoparticles**
					Polymeric nanogels Core/shell nanoparticles Polymeric micelles Dendrimers
					**Inorganic nanoparticles**
					Silica nanoparticles Carbon nanotubes Magnetic nanoparticles Gold nanoparticles
					**Lipid nanoparticles**
					Nanoemulsions Microemulsions Liposomes Solid lipid nanoparticles Nanostructured lipid carriers

## Data Availability

Not applicable.
